# Sensitivity of alternative measures of functioning and wellbeing for adults with sickle cell disease: comparison of PROMIS® to ASCQ-Me℠

**DOI:** 10.1186/s12955-017-0661-5

**Published:** 2017-06-02

**Authors:** San Keller, Manshu Yang, Marsha J. Treadwell, Kathryn L. Hassell

**Affiliations:** 1 American Institutes for Research, 100 Europa Drive, Suite 315, Chapel Hill, NC 27517-2357 USA; 2 Children’s Hospital & Research Center Oakland, 747 52nd Street, Oakland, CA 94609 USA; 3 Division of Hematology, University of Colorado, 12700 E. 19th Avenue, Rm 9122 RC 2/MS B170, Aurora, CO 80045 USA

**Keywords:** ASCQ-Me℠, PROMIS®, Validity, Sickle cell disease, Patient-reported outcomes, Item response theory

## Abstract

**Background:**

Sickle Cell Disease (SCD) causes profound suffering and decrements in daily functioning. Demand is growing for valid and reliable measures to systematically document these effects, particularly in adults. The Adult Sickle Cell Quality of Life Measurement System, ASCQ-Me℠, was developed for this purpose. ASCQ-Me℠ is one of four measurement systems housed within the Person–Centered Assessment Resource (PCAR), funded by the National Institutes of Health, to support clinical research. To help users select the best of these measures for adults with SCD, we evaluated and compared two PCAR systems: one designed to be “universally applicable” (the Patient-Reported Outcome Measurement Information System, PROMIS®) and one designed specifically for SCD (ASCQ-Me℠).

**Methods:**

Respondents to PROMIS and ASCQ-Me questions were 490 adults with SCD from seven geographically-disbursed clinics within the US. Data were collected for six ASCQ-Me measures (Emotional Impact, Sleep Impact, Social Impact, Stiffness Impact, Pain Impact, SCD Pain Episode Frequency and Severity) and ten PROMIS measures (Pain Impact, Pain Behavior, Physical Functioning, Anxiety, Depression, Fatigue, Satisfaction with Discretionary Social Activities, Satisfaction with Social Roles, Sleep Disturbance, and Sleep-Related Impairment). Statistical analyses, including analysis of variance and multiple linear regression, were conducted to determine the sensitivity of measures to SCD severity. SCD severity was assessed via a checklist of associated treatments and conditions.

**Results:**

For those with the most severe SCD, PROMIS scores showed worse health compared to the general population for nine of ten health domains: the magnitude of the difference ranged 0.5 to 1.1 standard deviation units. The PROMIS domains most severely affected were Physical Functioning and Pain (Impact and Behavior). Significant differences by tertile of the SCD-MHC were shown for most PROMIS short forms and all ASCQ-Me short and fixed forms. In most models, ASCQ-Me measures explained statistically significant unique variance in SCD-MHC scores complementary to that explained by corresponding PROMIS measures.

**Conclusions:**

Study results supported the validity of both PROMIS and ASCQ-Me measures for use in adults with SCD. Compared to comparable PROMIS scores, most ASCQ-Me scores were better predictors of SCD disease severity, as measured by a medical history checklist. The clinical implications of these results require further investigation.

## Background

Sickle Cell Disease causes profound suffering and decrements in daily functioning [1, 2]. The Adult Sickle Cell Quality of Life Measurement System (ASCQ-Me℠, pronounced “Ask me”), was developed to address the growing demand for valid and reliable measures to systematically document these effects in adults [3]. ASCQ-Me℠ is one of four measurement systems housed within the Person–Centered Assessment Resource (PCAR), funded by the National Institutes of Health to support clinical research. [4, 5]. Of interest to users of these measures is evidence-based information regarding how and when to apply each. Here we report such a study describing the validity of the Patient-Reported Outcome Measurement Information System (PROMIS®) which was designed to be applicable across chronic diseases [6, 7], as well as one of the condition-specific measures included in the PCAR -- ASCQ-Me℠ [2]. The health assessments in both PROMIS and ASCQ-Me were built and scored using Item Response Theory (IRT), specifically the Graded Response Model (GRM), and both use a web-based, electronic data collection platform [3, 8]. Given that PROMIS was designed to be universally applicable, [9] the value added of a system like ASCQ-Me may be in question.

Sickle cell disease (SCD) is one of the most common genetic disorders in the USA affecting up to 100,000 individuals. [10] Adults with SCD face debilitating health problems including multi-organ failure, chronic pain and neurocognitive deficits. [11, 12] Adult care for patients with SCD lags pediatric care because SCD used to be a disease of childhood with few individuals living long enough to become adults. Now, with widely-adopted infant screening practices, major advances in therapy, and increased use of preventative medicine, the vast majority of individuals with SCD grow out of pediatric care [13–16]. Therefore, beginning in 2002, the National Heart, Lung and Blood Institute (NHLBI) conducted a series of workshops that focused on ways to improve treatment for adults living with SCD [17]. Stakeholders determined the need for a systematic, reliable and valid method for documenting adult patient-reported outcomes (PRO) for SCD which led to the creation of ASCQ-Me. A number of treatments currently are available to improve the functioning and wellbeing of adults with SCD [18–22]. To inform the choice of therapy, the effects of these alternative treatments need to be systematically documented using data that are comparable across studies. Research and development of ASCQ-Me was conducted during the same time period as that for PROMIS and, like PROMIS, used IRT to evaluate and calibrate questions for inclusion in the system [23, 24]. This enabled the development of a computer adaptive testing (CAT) system for ASCQ-Me [25].

There is a long-standing debate in the PRO literature regarding the relative advantages of generic assessments of functioning and well-being compared to condition-specific measures of the same [26]. Some research suggests that disease-specific indicators may lack relevance because many aspects of functioning (e.g. sleep, sexual, or cognitive functioning) and wellbeing (e.g. depression, pain, fatigue) are, in fact, not specific to a particular condition [27, 28]. Other research demonstrates that the amount of evidence available to interpret the meaning of a measure is increased when it is possible, as it is with generic measures, to accumulate data across conditions and treatments [29–31]. Finally, a generic approach to measuring functioning and wellbeing has a practical value because a new measurement system does not have to be created for every specific chronic condition: rather, the resources that would have gone into creating an alternative measure can be put into designing studies which go beyond measure development.

Yet, the practical value of generic measures is only relevant if their validity to assess outcomes for specific conditions is comparable to that of condition-specific measures. The evidence in this regard is inconsistent. Compared to generic measures, condition-specific measures have sometimes been shown to be more sensitive to differences in disease severity [32, 33], and sometimes less sensitive [34, 35]. For example, the Functional Assessment of Cancer Therapy-Colorectal PRO (a condition-specific measure) was found to be more responsive to change in condition for patients with colorectal cancer, than the Short Form-12 Health Survey version 2 (a generic measure) [33]. In contrast, the Short Form 36 Bodily Pain scale (a generic measure) was found to be more responsive to worsening of symptoms in a group of patients with diagnosis of herniated disc, spinal stenosis, and spondylosis, than two disease-specific measures (the Oswestry Diability Index or the MODEMS) [34]. A comparison between generic and condition-specific health-related quality of life in children with SCD showed that the condition-specific measure provided important information not provided by the generic measure [36].

There is also disagreement about the characteristics of measures that define them as either generic or condition specific. The *condition-attribution* approach is to take generic questions and modify them so that the respondent answers them only with regard to the condition [27, 37, 38]. Each item (e.g. “How severe is your pain?”) would have an attribution to the condition (e.g. “How severe is your sickle cell pain?”). Following this approach, condition-specific items can be formed by simply modifying existing questions. The condition attribution approach is efficient -- patient interview data would not have to be collected and analyzed in order to generate the condition-specific items. Yet, FDA guidelines on the development of PROs require that patient interviews be part of the development process [39] and this favors the *content-validity* approach to developing condition-specific measures. The content-validity approach is to base items on aspects of functioning and wellbeing that persons with the condition have spontaneously offered in semi-structured interviews or that are known features of the clinical presentation. That is, the content is condition-specific because it has been reported by persons with the condition [40–44] and this is the approach that we used to develop ASCQ-Me [2].

Previous research comparing the measurement properties of selected PROMIS item banks to condition-specific measures of the same or related domains, in general, has supported the use of PROMIS as an alternative to condition specific measures. PROMIS measures were shown to provide precise measurement over a broader range of scores on the latent trait than legacy measures [45, 46]. For example, in 17,726 patients with osteoarthritis, compared to arthritis-specific PROs (the Western Ontario and McMaster Universities Arthritis Index, WOMAC, and the Health Assessment Questionnaire, HAQ), the PROMIS Physical Functioning (PF) CAT scores had lower standard errors over a broader range of physical function latent trait scores [47]. The PROMIS PF CAT also was shown to be more sensitive to change in condition following knee surgery than either a condition-specific PRO (the International Knee Documentation Committee, IKDC, scale) or an electronic walking performance measure [35]. One reason PROMIS may perform well in these contexts is that the added precision of adaptive assessment makes up for any precision loss that may be due to PROMIS’ lack of condition-specific content. Indeed, a comparison of the PROMIS Depression CAT to a variety of fixed-length forms from the same item bank showed the CAT to be more precise and have lower ceiling and floor effects [48]. Thus, a more valid comparison of PROMIS to condition specific measures would keep the type of measure constant. That is, comparisons would be made between fixed format PROMIS measures and fixed format condition-specific measures or between PROMIS CATs and condition-specific CATs.

Here we compare the measurement properties of PROMIS and ASCQ-Me using fixed formats for each. Moreover, our earlier research [3] did not test the reliability and validity of ASCQ-Me fixed forms and so we provide this evidence as well. The objective of this research is to produce information useful to those interested in using either PROMIS or ASCQ-Me to assess outcomes for adults with SCD. Thus, we conducted a descriptive study to accomplish four tasks: (1) to publish evidence regarding the reliability and validity of the ASCQ-Me fixed forms and short forms (SFs), (2) to describe the precision of the ASCQ-Me fixed forms to discriminate among levels of SCD severity; (3) to describe the validity of PROMIS Version 1.0 SFs to assess health outcomes for adults with SCD, and (4) to determine which scores measuring similar health concepts provided the most information about SCD severity.

## Methods

### Participants

PROMIS and ASCQ-Me field test data were collected at seven geographically diverse sites with the assistance of site coordinators trained in a standardized study protocol. The targeted enrollment across sites was set to obtain sufficient sample size for the psychometric analyses (500 patients) assuming a ten-percent rate of no-shows; and we attempted to achieve diversity in age and gender. Eligible participants were adults with 18 years of age or older at the time of data collection and diagnosed with sickle cell disease. People who were younger than 18, did not have a diagnosis of SCD, had a diagnosis of sickle cell trait, or could not read English, were excluded from the study. We sought to be inclusive of the variability in adult patients who were seen in ambulatory clinics in the U.S., including those in steady state and on therapy, so we had no other exclusions. It is important to note that the same group of patients completed both ASCQ-Me and PROMIS questions so that any differences in the ASCQ-Me and PROMIS scores would not be attributable to differences in the people who provided the data for each.

### Measures

We required a method of identifying groups of patients who differed in their SCD severity in order to evaluate the ability of ASCQ-Me and PROMIS measures to reflect differences in suffering and functioning of people who differed in the extent of their disease. This was challenging because there is no consensus method for assessing SCD severity. SCD is characterized by the type of mutations to the pair of beta-hemoglobin (Hb) genes, variations include Hb-SS, Hb-SC and Hb-Sβ [49, 50] and individuals with Hb-SS usually, but not always, have more symptoms than those with other genotypes [51–53]. However, genotype is not a reliable indicator of disease severity because variation of symptomatology within genotypes is so broad [54–56]. Frequency of hospitalizations has been used as a marker of disease severity [57–61]; yet, other data indicate that a large percentage of patients who suffer from extreme pain never go to the hospital [62–64].

Nevertheless, adult sickle cell providers seeing a patient for the first time ask that patient a set of questions to gauge the severity of his or her disease. A medical history characterized by prescription pain medication, blood transfusions and a number of these diagnoses (i.e., retinopathy, avascular necrosis, leg ulcers, kidney disease, stroke, and pulmonary hypertension) in a person presenting with SCD could indicate severe disease [65–69]. In the absence of a consensus method for determining severity, we reasoned that a method which mimicked the clinical interview in content would identify patients who differed in the amount of SCD-related damage caused by their sickle cell, and could, thus, serve as a surrogate marker of disease severity. Following this logic we included a checklist of seven conditions usually secondary to SCD and two treatments indicative of severity as part of the data collection. For convenience, we refer to this indicator as the SCD Medical History Checklist (SCD-MHC).

In previous research this measure demonstrated discriminant validity with regard to a checklist of conditions not associated with SCD, convergent validity with alternative indicators of SCD severity (number and severity of vaso-occlusive incidents, frequency of emergency department visits in the past year), and resistance to common method bias [3]. The SCD-MHC was scored as the sum of the endorsed questions -- the method employed in previous research with such checklists [70–72] -- and supported by research showing negligible differences between unit and alternative weighting methods for the scoring of checklists [73, 74].

ASCQ-Me measures included five-item SFs for Emotional Impact, Sleep Impact, Social Impact, Stiffness Impact, Pain Impact; and a five-item, pain episode fixed form scored as Pain Episode Frequency (two items) and Pain Episode Severity (three items). We use the term “fixed form” to indicate that these are not adaptive measures because all respondents are presented with the same items in the same sequence. In contrast, all ASCQ-Me short forms are subsets of items from the corresponding ASCQ-Me item banks. The Pain Episodes items are not short forms because they are not drawn from the ASCQ-Me item banks, but they are fixed forms because the items are presented in a fixed sequence. PROMIS measures included version 1.0 SFs for Pain Impact, Pain Behavior, Physical Functioning, Anxiety, Depression, Fatigue, Satisfaction with Discretionary Social Activities, Satisfaction with Social Roles, Sleep Disturbance, and Sleep-Related Impairment. PROMIS SFs ranged in length from six to ten questions; with most (eight out of ten SFs) containing either seven or eight questions.

Table 1 denotes the PROMIS measures that correspond to each of the ASCQ-Me measures and shows that for each ASCQ-Me measure (except for Stiffness Impact), there were two corresponding PROMIS measures. PROMIS Fatigue does not have a corresponding ASCQ-Me SF. Table 1 also describes the differences in the scoring for ASCQ-Me and PROMIS scales.

**Table 1:** ASCQ-Me fixed and short forms^a^, corresponding PROMIS short forms, direction of scoring for each

ASCQ-Me	Higher scores mean	PROMIS^b^	Higher scores mean
Emotional impact	Better health	Anxiety; Depression	More suffering
Pain impact	Better health	Pain Interference; Pain Behavior	More suffering
Pain episodes^**c**^	More suffering	Pain Interference; Pain Behavior	More suffering
Sleep impact	Better health	Sleep Disturbance; Sleep-Related Impairment	More suffering
Social functioning impact	Better health	Satisfaction with Social Activities; Satisfaction with Social Roles	Better health
Stiffness impact^**d**^	Better health	Physical Functioning	Better health

^a^We use the term “fixed form” to indicate that these are not adaptive measures because all respondents are presented with the same items in the same sequence. All ASCQ-Me short forms are subsets of items from the corresponding ASCQ-Me item banks. The Pain Episodes items are not short forms because they are not drawn from the ASCQ-Me item banks, but they are fixed forms because the items are presented in a fixed sequence

^b^When more than one PROMIS measure corresponds to an ASCQ-Me measure, each is separated by a semi-colon

^c^The Pain Episodes measure includes two subscales to assess frequency and severity

^d^PROMIS does not have a stiffness measure but stiffness is related to physical ability

To be consistent with widely-used health status measures [75–77], most ASCQ-Me scores are calculated in the direction of overall health such that higher ASCQ-Me scores indicate better health. The one exception is the Pain Episodes measure for which higher scores mean more frequent and severe pain episodes. PROMIS scores for health concepts that describe functioning (e.g. physical and social functioning) are scored in this direction as well (higher scores indicate better health). PROMIS scores for symptom burden (e.g. depression, sleep problems, pain) are calculated such that higher scores indicate poorer health, consistent with symptom burden. These differences do not affect statistical analysis of variance attributable to each measure. However, in comparing ASCQ-Me and PROMIS with regard to associations between scores and criterion variables, these differences must be kept in mind. For example, the correlation between ASCQ-Me measures of symptoms and PROMIS measures of symptoms will be negative because lower scores on the PROMIS measures indicate less of the symptom while higher scores on the ASCQ-Me measures indicate less of the symptom.

Both ASCQ-Me and PROMIS are scored so that “50” is the average for the population on which their questions were calibrated; and 10 points is equivalent to one standard deviation in that population [30]. For ASCQ-Me, scores were based on the 556 adults with SCD who participated in the field test [3]. The sociodemographic characteristics of this population were consistent with the sociodemographic characteristics of the adult clinical population who have SCD [78, 79]. For PROMIS, scores were based on a sample from the general US population and included individuals with and without chronic disease [30, 80].

### Data collection procedure

Patients signed a consent form after they arrived at one of ASCQ-Me field test sites. They were then seated at a computer and a site coordinator helped them to log onto the ASCQ-Me website. Sites confirmed that participants had SCD. The site coordinator entered the SCD type, and assisted the respondent in reviewing a tutorial that demonstrated how to navigate through the survey. Respondents completed ASCQ-Me questions first, took a 30-min break if they wanted one, and then completed the PROMIS measures. Respondents received an honorarium for their participation. We limited our analytic sample to the 490 respondents who had completed both ASCQ-Me and PROMIS assessments.

### Analytic methods -- reliability and validity of ASCQ-Me short- and fixed forms

Reliability and validity evidence has been published for the ASCQ-Me item banks [3] and for the PROMIS measures [80, 81] but not for the ASCQ-Me SFs. The ASCQ-Me SF scoring algorithms incorporated IRT item calibrations but we present internal consistency reliability estimates using coefficient alpha [82] rather than test information curves to facilitate interpretation of the results and to enable audiences to compare our estimates of reliability to those available for other measures which report alpha. Construct validity of the ASCQ-Me SFs and pain episode fixed forms was assessed by examining the correlations of short-form scores to item-bank scores for the same health concepts and to PROMIS SFs for similar concepts. As explained in Table 1, in many cases there is more than one PROMIS score corresponding to a particular ASCQ-Me score. When this is the case, the range of correlations among the ASCQ-Me score and the PROMIS scores will be presented. Construct validity was also evaluated by determining the ability of the SF scores to discriminate among groups of participants formed on the basis of their SCD-MHC scores -- representing low, medium, and high levels of severity. Low, medium and high cut-offs for the SCD-MHC were based on tertiles of the distribution of scores. SCD-MHC scores were the sum of the number conditions checked. Cutoffs for low, medium and high groups were SCD-MHC scores less than 2, equal to 2, and greater than 2, respectively.

### Analytic methods - precision of ASCQ-Me and PROMIS to discriminate among levels of SCD severity

We calculated the average score for respondents within each tertile of SCD severity for all of the ASCQ-Me and PROMIS measures and created histograms to examine the pattern of scores. We examined these patterns to determine: 1) whether there was a monotonic relationship between levels of SCD severity and mean scores on the ASCQ-Me and PROMIS measures; 2) which SFs indicated a decrease in health corresponding to an increase in SCD severity, and 3) whether the patterns of relationships between SCD severity and health for similar health concepts was similar for ASCQ-Me and PROMIS. We used univariate analysis of variance (ANOVA) to test for differences in means among levels of SCD severity with a Bonferroni correction to the significance level to account for the family-wise error rate (i.e. 0.05/17 = 0.0029) [83]. The relative precision of each scale was described by dividing the F-statistic associated with each scale by the largest F-statistic in the group [84, 85].

### Analytic methods- comparative sensitivity of PROMIS and ASCQ-measures of similar health concepts

These analyses provided information about the amount of unique variance in SCD severity accounted for by ASCQ-Me compared to PROMIS. We fitted multiple linear regression models to evaluate the relationship of ASCQ-Me and PROMIS scores to SCD-MHC scores controlling for the effect of sex, age and genotype. For this set of analyses, SCD-MHC scores were left continuous. We used Type III sum of squares to compute the F-statistic for the unique variance in SCD-MHC associated with ASCQ-Me and PROMIS scores. Three models each were fitted to compare the effects of the PROMIS Pain Impact and Behavior SFs to three ASCQ-Me pain measures (Pain Impact, Pain Episode Frequency, and Pain Episode Severity). We applied the Bonferroni correction to the significance level of statistics from these models to account for the family-wise error rate (i.e. 0.05/3 = 0.0166). Two models each were fitted to compare the effect of ASCQ-Me scores to two corresponding PROMIS measures for the emotional, social and sleep domains. The significance level for the associated statistics was set at 0.025 (i.e. 0.05/2 = 0.025).

## Results

### Respondent characteristics

A total of 490 adults completed both the ASCQ-Me and PROMIS questions. Of these, just 6.5% of participants were older than 55; and roughly 30% were in each of the age ranges of 18–24 (30%), 25–34 (33%), and 35–54 (31%). Nearly two-thirds of respondents were female (64%). Almost two-thirds of respondents had Sickle Cell Anemia (Hemoglobin SS - 65%) and the rest either had Sickle Hemoglobin C Disease (Hemoglobin SC - 20%), Sickle Beta-Thalassemia Disease (Hemoglobin S Beta Plus or Zero Thalassemia 10%) or Sickle Cell type unspecified (5%).

### ASCQ-Me fixed form reliability and validity

Table 2 shows the psychometric properties of the five ASCQ-Me SFs. Cronbach’s alpha coefficients for all ASCQ-Me SFs were well above 0.90 and latent health scores obtained from SFs had a very high correlation with those obtained from full item banks (>0.95 in every case).

**Table 2:** Reliability and validity of ASCQ-Me fixed and short forms^a^

	Number of items	Cronbach’s Alpha	Full ASCQ-Me item bank	Similar PROMIS short form(s)^b^
ASCQ-Me short forms				
Emotional impact	5	0.901	0.96	−0.69 to −0.73^c^
Pain impact	5	0.942	0.99	−0.72 to −0.80
Sleep	5	0.930	0.98	−0.54 to −0.80
Social	5	0.921	0.98	0.58 to 0.65
Stiffness	5	0.918	0.97	0.64
ASCQ-Me pain episode fixed forms				
Pain episode frequency	2	0.799	−0.54^d^	0.42 to 0.47
Pain episode severity	3	0.727	−0.26	0.26 to 0.26

^a^We use the term “fixed form” to indicate that these are not adaptive measures because all respondents are presented with the same items in the same sequence. All ASCQ-Me short forms are subsets of items from the corresponding ASCQ-Me item banks. The Pain Episodes items are not short forms because they are not drawn from the ASCQ-Me item banks, but they are fixed forms because the items are presented in a fixed sequence

^b^There is more than one PROMIS score to correspond to the first five ASCQ-Me scores (see Table 1) and for those, there will be a range of correlations reported. These correlations are negative consistent with differences between the way ASCQ-Me and PROMIS scores are calculated

^c^PROMIS measures of symptoms (i.e. anxiety, depression, pain, sleep disturbance) are scored such that higher scores mean more of the symptom; whereas, all of the ASCQ-Me measures – with the exception of the pain episodes – are scored such that higher scores mean better health. The negative correlation reflects the difference in the direction of scoring

^d^The negative correlation between the ASCQ-Me Pain Episode measures and the ASCQ-Me Pain Impact item bank is an artifact of the direction of scoring: a high score for Pain Episodes means *more* pain whereas a high score for the Pain Impact item bank means *less* pain

All correlations between ASCQ-Me SFs and PROMIS SFs for similar health concepts were large (>0.50) [86] and ranged from 0.80 to 0.54 with a median correlation of 0.69. The ASCQ-Me Emotional Impact scores had a stronger correlation with the PROMIS Depression scores than with the PROMIS Anxiety scores. The ASCQ-Me Pain Impact scores were more strongly related to the PROMIS Pain Interference than to the PROMIS Pain Behavior scores.

Evidence for the reliability of the ASCQ-Me pain episode fixed forms was not as strong, although internal consistency reliabilities exceeded 0.70 -- a frequently used rule of thumb for evaluating whether a measure is reliable enough for use in statistical comparisons at the group level [87]. Of the two, the ASCQ-Me Pain Episode *Frequency* measure had higher internal consistency reliability and a stronger relationship to the ASCQ-Me Pain Impact item bank and to the PROMIS Pain Interference and the PROMIS Pain Behavior SFs. Correlations between the ASCQ-Me pain episode fixed forms and alternative assessments of pain were not as large as those between the ASCQ-Me pain short form and the PROMIS pain measures. We address this in the discussion.

Taken together, these results support both the reliability and validity of the ASCQ-Me SF scores, in particular, and suggest that the results found for the SFs would be indicative of results that would be obtained with the full item bank. Other evidence for the validity of the ASCQ-Me SFs is presented below.

Table 3 displays the mean scores for seven ASCQ-Me and ten PROMIS fixed format measures at each level of SCD severity. The measures are ordered in terms of the differences in the means among level of severity starting with the measure that differs the most across levels of severity.

**Table 3:** Discrimination of ASCQ-Me and PROMIS scores among levels of SCD severity

ASCQ-Me & PROMIS measures	# Items	Low	Medium	High	F-Stat^a^	df^b^ error/total
ASCQ-Me Stiffness Impact	5	53.80	48.81	45.81	38.07***	485/487
PROMIS Physical Functioning	10	45.75	43.22	38.70	34.64***	483/485
PROMIS Pain Impact	6	53.83	57.46	60.69	29.61***	482/484
ASCQ-Me Pain Impact	5	53.26	48.88	46.30	28.06***	486/488
PROMIS Pain Behavior	7	53.46	57.06	60.57	27.47***	485/487
PROMIS Social Roles	7	49.27	45.89	43.26	19.79***	485/487
ASCQ-Me Social Impact	5	52.72	48.91	46.72	18.21***	473/475
PROMIS Social Activities	7	51.83	49.29	47.08	12.78***	484/486
ASCQ-Me Emotional Impact	5	51.84	49.75	47.04	11.81***	483/485
ASCQ-Me Sleep Impact	5	52.24	48.79	48.10	10.42***	487/489
PROMIS Fatigue	7	54.41	55.50	58.24	9.34***	484/486
PROMIS Depression	8	51.93	53.29	56.15	8.42**	485/487
PROMIS Sleep Disturbance	8	52.41	55.20	56.57	7.70**	486/488
ASCQ-Me Pain Episode Severity	3	48.38	50.09	52.10	6.38*	487/489
ASCQ-Me Pain Episode Freq.	2	48.31	50.33	51.99	6.32*	487/489
PROMIS Anxiety	7	52.27	53.49	55.04	4.24	486/488
PROMIS Sleep Impairment	8	53.37	53.92	55.34	1.93	486/488

^a^F-statistic associated with one-way analysis of variance

^b^Degrees of freedom (df). For the model this is always equal to 2

***p < 0.0001; **p < 0.001; *p < 0.003

The scores which discriminated most among levels of SCD severity were the ASCQ-Me Stiffness Impact, PROMIS Physical Functioning and ASCQ-Me and PROMIS pain SF measures. Next most discriminating were the ASCQ-Me and PROMIS social functioning, the ASCQ-Me Sleep and Emotional impact, and the PROMIS Fatigue scores. Among the least discriminating were the PROMIS sleep and emotional and the ASCQ-Me pain episode scores. For all but the PROMIS Anxiety and Sleep Impairment measures, the probability associated with differences in the means for all of the ASCQ-Me and eight of ten of the PROMIS measures was less than the cut-off of 0.0029 for statistical significance (see Table 3). In Table 4 we present these results separately for PROMIS and ASCQ-Me measures to facilitate comparisons of the sensitivity of different scores within each measurement system.

**Table 4:** Discrimination of ASCQ-Me and PROMIS scores: within system comparison

	# Items	Low	Medium	High	F-Stat^a^	df^b^ error/total
ASCQ-Me measures						
ASCQ-Me stiffness impact	5	53.80	48.81	45.81	38.07***	485/487
ASCQ-Me Pain Impact	5	53.26	48.88	46.30	28.06***	486/488
ASCQ-Me Social Impact	5	52.72	48.91	46.72	18.21***	473/475
ASCQ-Me Emotional Impact	5	51.84	49.75	47.04	11.81***	483/485
ASCQ-Me Sleep Impact	5	52.24	48.79	48.10	10.42***	487/489
ASCQ-Me Pain Episode Severity	3	48.38	50.09	52.10	6.38*	487/489
ASCQ-Me Pain Episode Freq.	2	48.31	50.33	51.99	6.32*	487/489
PROMIS measures						
PROMIS Physical Functioning	10	45.75	43.22	38.70	34.64***	483/485
PROMIS Pain Impact	6	53.83	57.46	60.69	29.61***	482/484
PROMIS Pain Behavior	7	53.46	57.06	60.57	27.47***	485/487
PROMIS Social Roles	7	49.27	45.89	43.26	19.79***	485/487
PROMIS Social Activities	7	51.83	49.29	47.08	12.78***	484/486
PROMIS Fatigue	7	54.41	55.50	58.24	9.34***	484/486
PROMIS Depression	8	51.93	53.29	56.15	8.42**	485/487
PROMIS Sleep Disturbance	8	52.41	55.20	56.57	7.70**	486/488
PROMIS Anxiety	7	52.27	53.49	55.04	4.24	486/488
PROMIS Sleep Impairment	8	53.37	53.92	55.34	1.93	486/488

^a^F-statistic associated with one-way analysis of variance

^b^Degrees of freedom (df). For the model this is always equal to 2

***p < 0.0001; **p < 0.001; *p < 0.003

### Precision of ASCQ-Me and PROMIS to discriminate among levels of SCD severity

**Fig. 1:**
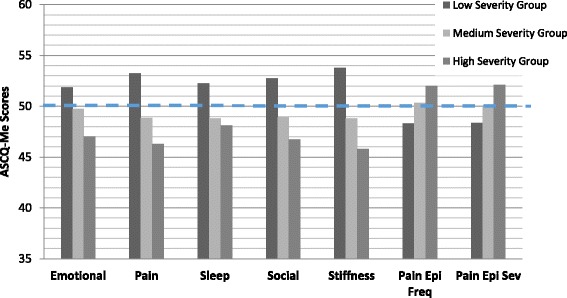
ASCQ-Me scores at *low*, *medium* and *high* SCD severity. The ASCQ-Me measures shown on the X-axis are: *Emotiona*l Impact (Emotional); *Pain* Impact (Pain); *Sleep* Impact (Sleep); *Social* Impact (Social); *Stiffness* Impact (Stiffness); SCD *Pain Episode Frequency* (Pain Epi Freq); SCD *Pain Episode* Severity (Pain Epi Sev). The Pain Episode measures are scored such that a higher score indicates more pain whereas the other ASCQ-Me measures are scored so that a higher score means better health

The histograms in Fig. 1 show the monotonic relationship between the three means for each ASCQ-Me measure corresponding to the respondent SCD severity grouping of low, medium and high, respectively. In every case, those with ASCQ-Me scores indicating the worst health were found in the highest tertile of SCD severity. The thick, dashed, horizontal line which intersects the vertical axis at 50 represents the average score in the ASCQ-Me field test sample, indicating that those in the top and bottom tertiles of the SCD-MHC, had ASCQ-Me scores showing poorer and better than average health, respectively.

**Fig. 2:**
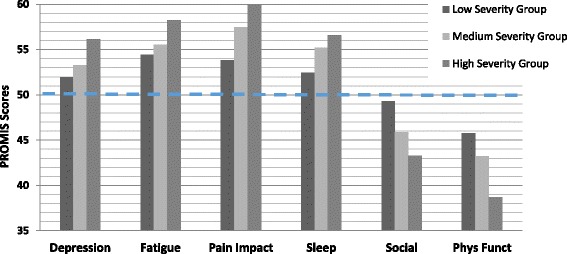
PROMIS scores at *low*, *medium* and *high* SCD severity. The PROMIS measures shown on the X-axis are: *Depression; Fatigue; Pain Impact; Sleep* *Disturbance (Sleep); Satisfaction with Social Roles (Social); Physical Functioning (Phys Funct)*. The PROMIS Social and Physical Function measures are scored so that a higher score means more functioning and better health; whereas the other measures are scored so that a higher score means more suffering and poorer health

There were two PROMIS SFs each for domains of pain impact, emotional impact, sleep impact, and social impact (see Table 2) and we display the histograms in Fig. 2 for the domain short form which had the strongest relationship to SCD severity. Thus, we did not include the histograms for PROMIS Pain Behavior, Anxiety, Sleep-Related Impairment, and Satisfaction with Discretionary Social Activities in Fig. 2, but the means for these measures at each level of SCD severity can be found in Table 3. All PROMIS measures displayed a monotonic relationship with mean scores systematically showing better health at lower levels of SCD severity. With the general population mean of 50 as the reference line (see thick dashed line at 50 on the graph), these graphs show that adults with SCD, even those with less severe disease, were always less healthy than the general population across all PROMIS measures. Recall that one standard deviation unit on PROMIS and ASCQ-Me metrics is equivalent to 10 points. Those with the most severe disease scored around one standard deviation worse than the general population and even those with the least severe disease scored nearly half as standard deviation worse than the general public on the PROMIS Physical Functioning, Fatigue and Pain Impact SFs.

### Comparative sensitivity of PROMIS and ASCQ-Me scores to variability in SCD severity

Table 5 describes the unique variance in SCD severity explained by ASCQ-Me and PROMIS scores after controlling for the effects of age, sex, and SCD genotype. Pain is the hallmark symptom of SCD; thus, we compared the information value of all of the ASCQ-Me and PROMIS pain measures (results for first six models, top half of the table).

**Table 5:** Comparison of unique variance in SCD severity explained by ASCQ-Me and PROMIS measures

Model^a^		Type III test statistics	
		F	P^b^
ASCQ-Me & PROMIS measures on pain			
I	ASCQ-Me Pain Impact^d^	10.78	<0.0011
	PROMIS Pain Impact	3.15	0.0766
II	ASCQ-Me Pain Impact^d^	13.15	0.0003
	PROMIS Pain Behavior	5.72	0.0172
III	PROMIS Pain Impact^d^	33.96	<0.0001
	ASCQ-Me Pain Episode Frequency	1.16	0.2820
IV	PROMIS Pain Impact^d^	40.93	<0.0001
	ASCQ-Me Pain Episode Severity	5.27	0.0221
V	PROMIS Pain Behavior^d^	33.07	<0.0001
	ASCQ-Me Pain Episode Frequency	0.92	0.3381
VI	PROMIS Pain Behavior^d^	39.63	<0.0001
	ASCQ-Me Pain Episode Severity	5.75	0.0169
ASCQ-Me & PROMIS measures of similar health domains			
I	ASCQ-Me Stiffness Impact^d^	27.40	<0.0001
	PROMIS Physical Functioning^d^	6.92	0.0088
II	ASCQ-Me Social Impact^d^	8.17	0.0045
	PROMIS Satisfaction with Social Roles^d^	5.63	0.0181
III	ASCQ-Me Social Impact^d^	17.07	<0.0001
	PROMIS Satisfaction with Social Discretionary Activities	0.84	0.3594
IV	ASCQ-Me Emotional Impact^d^	6.94	0.0087
	PROMIS Depression	1.50	0.2214
V	ASCQ-Me Emotional Impact^d^	13.10	0.0003
	PROMIS Anxiety	0.04	0.8386
VI	ASCQ-Me Sleep Impact^d^	7.84	0.0053
	PROMIS Sleep Disturbance	0.25	0.6194
VII	ASCQ-Me Sleep Impact^d^	14.24	0.0002
	PROMIS Sleep Impairment	1.22	0.2694

^a^All regression models controlled for gender, age, and SCD genotype

^b^The criterion for significance was set at α = 0.05/3 = 0.0166 to control for the family-wise error rate given that the same measure is involved in three or fewer tests (e.g. PROMIS Pain Impact and Pain Behavior are involved in three tests)

^c^The criterion for significance was set at α = 0.05/2 = 0.025 to control for the family-wise error rate given that the same measure is involved in two or fewer tests (e.g. ASCQ-Me Social, Emotional and Sleep impact are involved in two tests)

^d^Statistically significant unique variance associated with SCD severity after correction for multiple comparisons

The results showed that, in comparison to the ASCQ-Me Pain Impact SF, the PROMIS Pain Interference and Pain Behavior SFs explained less unique variance in SCD severity (see results for Models I and II). By contrast, the PROMIS Pain Interference and Behavior scores explained more unique variance in SCD severity compared to the ASCQ-Me pain episode scores (see results corresponding to Models III-VI). The results for the second set of models (I-VII, bottom half of the table) show that compared to PROMIS scores for similar health domains, ASCQ-Me scores consistently explained more unique variance in SCD severity. In every case, the amount of unique variance explained by ASCQ-Me was statistically significant and the amount of unique variance explained by the PROMIS Physical Functioning and Satisfaction with Social Roles SFs was also statistically significant (see Models I and II, bottom part of the Table).

## Discussion

### Interpretation of the results – reliability and validity of ASCQ-Me fixed forms

ASCQ-Me fixed and short forms were shown to be highly reliable. The SFs, based on a subset of items from the full item banks, had internal consistency reliability coefficients ranging from 0.94 to 0.90, supporting their clinical use at the individual-patient level [88, 89]. The fixed-form pain episode measures had internal consistency reliabilities of 0.80 and 0.73 demonstrating good precision for use in group-level clinical research [88–90].

Results supported the construct validity of the ASCQ-Me fixed and short forms as well. The correlations between the ASCQ-Me SFs and the corresponding ASCQ-Me item bank, were very large and ranged from 0.96 to 0.99. These correlations support the use of the ASCQ-Me fixed forms as a substitute for the ASCQ-Me item banks. Correlations between the ASCQ-Me Pain Episode Frequency and Severity fixed forms and the full ASCQ-Me Pain Impact item bank were lower but still large to moderate (0.54 and 0.26 in absolute magnitude). The lower correlations suggest that the ASCQ-Me pain episode fixed forms measure aspects of pain which are not covered by the ASCQ Pain Impact SF or item bank. Indeed, a comparison of the content of the measures reveals important differences. There is no overlap in items between the ASCQ-Me pain episode measures and the ASCQ-Me Pain Impact item bank and the pain episode questions refer specifically to pain episodes rather than to pain in general. Moreover, the ASCQ-Me pain episode questions differ from the ASCQ-Me and PROMIS pain short-form questions because they refer to a different time frame. The ASCQ-Me pain episode questions refer either to the past 12 months or to ever; whereas the ASCQ-Me and PROMIS short form questions refer to the past 7 days. The lower correlation of the pain episode scores with the ASCQ-Me Pain Impact item bank also could be due, in part, to the comparatively lower reliability of the pain episode measures. All things being equal, a more reliable measure will have a higher correlation with a criterion than a less reliable measure [90].

ASCQ-Me scores were strongly related to SCD severity providing additional evidence of their construct validity. There was a consistently monotonic relationship between levels of SCD severity and ASCQ-Me, such that ASCQ-Me scores indicated worse health at higher levels of SCD severity. In every case, ASCQ-Me scores significantly discriminated among groups of patients defined by tertiles of SCD disease severity. The ASCQ-Me short-form scores demonstrated a stronger relationship to disease severity than did the ASCQ-Me pain episode scores. Those ASCQ-Me SFs most strongly related to SCD severity were the ASCQ-Me Stiffness, Pain, and Social Impact measures, respectively.

### Interpretation of the results –validity of PROMIS short forms

PROMIS scores were strongly related to SCD severity and there was a consistently monotonic relationship between levels of SCD severity and PROMIS scores wherein PROMIS indicated worse health at higher levels of SCD severity. PROMIS scores for these SCD patients, even at the lowest level of SCD severity, indicated impairment relative to the general population. This finding is consistent with the clinical picture of SCD as incurring some suffering and disability even among those with less symptomatic disease [16, 91, 92] and, thus, supports the validity of PROMIS as a measure of functional deficits and suffering for SCD. The PROMIS scores showing the most profound effect of SCD relative to the general population were Physical Functioning, Pain Interference, Pain Behavior, and Fatigue.

### Interpretation of results – comparisons among ASCQ-Me and PROMIS scores

One-way ANOVA models demonstrated that, whether measured by PROMIS or ASCQ-Me, ability to function physically, pain, and the ability to engage in social roles and activities were most affected by SCD severity (see last two columns of Table 3). The PROMIS Fatigue SF was highly, significantly related to SCD severity, although less so than the ASCQ-Me and PROMIS measures of physical and social function and pain. On the other hand, compared to PROMIS, ASCQ-Me SFs demonstrated a greater effect of disease severity on emotional distress and sleep. ASCQ-Me pain episode scores were not as sensitive to SCD severity as scores yielded by other ASCQ-Me measures. Taken together, these results support the validity of many of the PROMIS and ASCQ-Me SFs of similar health concepts to describe differences in disease severity.

In choosing among ASCQ-Me and PROMIS assessments of similar health concepts, one would want to compare the amount of unique information about disease severity that each provides. We used multiple linear regression models of the relationship of ASCQ-Me and PROMIS SFs to SCD severity holding constant the potentially confounding effects of age, gender and genotype (Table 4). These results consistently demonstrated that, compared to PROMIS SFs, ASCQ-Me SF scores of similar concepts explained more *unique* variance in SCD severity and did so with fewer items.

Still, we are left to wonder why ASCQ-Me scores were sometimes found to be more sensitive than PROMIS which measured similar health concepts. It is not because the ASCQ-Me questions ask respondents to attribute symptoms or functioning to SCD, because the results were found for scores based on questions that did not refer to SCD. Some prior research has shown better sensitivity for PROMIS scores to condition severity, when those scores were based on calibrations derived from patients with that specific condition [93]. So, the greater sensitivity of ASCQ-Me scores could be due to the items having been calibrated on an SCD sample. In prior research, we replicated the regression analyses described earlier using two types of scoring: IRT scoring (with weights determined by the GRM for both) and raw scoring (with unit weights). The pattern of differences between ASCQ-Me and PROMIS was largely the same regardless of scoring method, suggesting that the greater sensitivity of ASCQ-Me was due to the content originating in qualitative research with SCD patients, rather than the calibration sample [94].

### Limitations

The implications of these results are restricted by variables included in the data collection. The PROMIS suite of measures includes multiple short forms for each health concept – for example, there are 10 PROMIS SFs that assess physical functioning. We do not know whether these results would generalize to other SFs; however the versions used in this study are the ones in widest circulation. In addition, our condition severity data was self-reported. This research would be strengthened were a consensus “gold standard” method of measuring SCD severity available to define the low, medium and high severity groups. Because they derive from the same source, the relationship between our indicator of condition severity (SCD-MHC) to ASCQ-Me or PROMIS scores might be artifacts of the data collection method. This theory is not supported, however, by evidence of discriminant validity for the SCD-MHC in relationship to self-reports of conditions which are not related to SCD [3]. Time was another variable missing from the data collection. Data were cross-sectional, so we could not address the relationship of ASCQ-Me or PROMIS scores to change in condition.

The implications of these results also are restricted by the characteristics of the participants. Those older than 54 and with SCD Type other than SS were in the minority, so sample size prevented us from being able to evaluate the generalizability of these results to the elderly and those with genotypes other than SS. Implications are also restricted by the study participant background data we were able to obtain. For example, we did not have data on all the various therapies to which individuals had been exposed so we could not evaluate the generalizability of results to subsets of patients defined by therapy. While data came from seven geographically dispersed clinics throughout the country, we do not know how representative our field test sample is because a nationally-representative, descriptive study of the socio-demographic and health characteristics of adults with SCD does not yet exist. However, available data suggests that the characteristics of our sample are likely to mirror those of the other populations with regard to age and hemoglobin type, although males may have been under-represented [95–97].

### Future research

Sickling hemoglobin may cause obstruction of blood flow in the brain and so cognitive functioning is an important health domain for SCD [12]. Unfortunately, the cognitive functioning item bank that we developed for the ASCQ-Me field test did not demonstrate good psychometric properties and so it is not included among the ASCQ-Me measures approved for use [3]. Future research could be designed to collect the data to evaluate the validity of the PROMIS Cognitive Functioning measures in adults with SCD.

Future research also could be conducted to evaluate alternative PROMIS short forms. PROMIS was developed to provide banks of items from which clinicians and researchers could select subsets particularly relevant for their purposes. PROMIS short-forms created by selecting a subset of items from corresponding item banks using mixed methods, may result in more precise measures for use in SCD than either generic short forms or ASCQ-Me measures. Such research has been successful in developing PROMIS short forms for use in multiple sclerosis and fibromyalgia, for example [97, 98].

Research is being conducted to determine whether these fixed-form results would generalize to results based on ASCQ-Me and PROMIS CATs. We did not compare the sensitivity of the PROMIS CATs to the ASCQ-Me CATs because the field test did not administer the ASCQ-Me CAT. Evaluations of this using simulated ASCQ-Me CAT data [99] are under way.

These results have implications for the sample size requirements to achieve a certain level of statistical power -- a measure which yields a more precise score than another measure will require fewer respondents. But statistical differences are not the same as clinically meaningful differences. A clinically meaningful difference is one large enough to be perceived by patients and/or their providers and/or one which has implications for planning care [100]. Future studies are required to determine whether the differences in precision between ASCQ-Me and PROMIS scores have any consequences for clinical care.

Other research to link ASCQ-Me scores to PROMIS scores is underway so that ASCQ-Me scores can be understood in comparison with the general population. But future research is required to compare the responsiveness of ASCQ-Me and PROMIS scores to change in condition severity.

## Conclusions

Study results showed support for the validity of eight PROMIS SFs and all ASCQ-Me SFs and fixed forms to assess health outcome in adults with SCD. Compared to comparable PROMIS scores, most ASCQ-Me scores were better predictors of disease severity. The clinical implications of these results require further investigation. Future research also should evaluate the validity of PROMIS cognitive functioning measures for use in adults with SCD and the sensitivity of both PROMIS and ASCQ-Me measures to change in SCD severity over time.

## Abbreviations

ASCQ-Me: the Adult Sickle Cell Quality of Life Measurement System
CAT: Computer Adaptive Testing
FDA: the Food and Drug Administration
GRM: the Graded Response Model
HAQ: the Health Assessment Questionnaire
IKDC: the International Knee Documentation Committee
IRT: Item Response Theory
NHLBI: the National Heart, Lung and Blood Institute
NIH: National Institutes of Health
PCAR: Person–Centered Assessment Resource
PF: Physical Functioning
PRO: Patient reported outcome
PROMIS: the Patient-Reported Outcome Measurement Information System
SCD: Sickle Cell Disease
SCD-MHC: the SCD Medical History Checklist
SF: Short Form
WOMAC: the Western Ontario and McMaster Universities Arthritis Index

## References

[1] Claster S, Vichinsky EP (2003). Managing sickle cell disease. BMJ.

[2] Treadwell MT (2013). Adult sickle cell quality of life measurement information system (ASCQ-Me): conceptual model based on review of the literature and formative research. Clin J Pain.

[3] Keller SD (2014). Patient reports of health outcome for adults living with sickle cell disease: development and testing of ASCQ-Me item banks. Health Qual Life Outcomes.

[4] N.I.H. Department of Health and Human Services (2013). RFA-CA-13-008: Person-centered outcomes research resource.

[5] Smith A, et al. News from the NIH: Person-centered outcomes measurement: NIH-supported measurement systems to evaluate self-assessed health, functional performance, and symptomatic toxicity. Transl Behav Med. 2016;6(3):470–74. 10.1007/s13142-015-0345-9 PMC4987601 27528535

[6] Fries JF, Bruce B, Cella D (2005). The promise of PROMIS: using item response theory to improve assessment of patient-reported outcomes. Clin Exp Rheumatol.

[7] DeWalt DA (2007). Evaluation of item candidates: the PROMIS qualitative item review. Med Care.

[8] Cella D (2007). The future of outcomes measurement: item banking, tailored short-forms, and computerized adaptive assessment. Qual Life Res.

[9] Adler D (2007). Developing the Patient-Reported Outcomes Measurement Information System (PROMIS). Med Care.

[10] Aisiku IP (2009). Comparisons of high versus low emergency department utilizers in sickle cell disease. Ann Emerg Med.

[11] Brawley OW (2008). National Institutes of Health Consensus Development Conference statement: hydroxyurea treatment for sickle cell disease. Ann Intern Med.

[12] DeBaun MR, Kirkman FJ. Central nervous system complications and management in sickle cell disease. Blood. 2016;127(7):829–38. 10.1182/blood-2015-09-618579 26758917

[13] Quinn CT (2010). Improved survival of children and adolescents with sickle cell disease. Blood.

[14] DeBaun MR, Telfair J (2012). Transition and sickle cell disease. Pediatrics.

[15] Halasa NB (2007). Incidence of invasive pneumococcal disease among individuals with sickle cell disease before and after the introduction of the pneumococcal conjugate vaccine. Clin Infect Dis.

[16] Yawn BP (2014). Management of sickle cell disease: summary of the 2014 evidence-based report by expert panel members. Jama.

[17] National Heart, Lung and Blood Institute [website]. Workshop on Adults with Sickle Cell Diseases: Meeting Unmet Needs. Executive Summary and Recommendations. Retrieved September 8, 2015, from http://www.nhlbi.nih.gov/meetings/scdmtng/execsum.htm.

[18] Badawy SM, et al. Health-related quality of life and adherence to hydroxyurea in adolescents and young adults with sickle cell disease. Pediatr Blood Cancer. 2016 Nov.;28 [Epub ahead of print]. 10.1002/pbc.26369 27896936

[19] Beverung LM (2015). Health-related quality of life in children with sickle cell anemia: impact of blood transfusion therapy. Am J Hematol.

[20] Bhatia M (2015). Health-related quality of life after allogeneic hematopoietic stem cell transplantation for sickle cell disease. Biol Blood Marrow Transplant.

[21] Kelly MJ (2012). Journeys to recovery study, HSCT-CHESS™ study. Health-related quality of life (HRQL) in children with sickle cell disease and thalassemia following hematopoietic stem cell transplant (HSCT). Pediatr Blood Cancer.

[22] Thornburg CD (2011). Differences in health-related quality of life in children with sickle cell disease receiving hydroxyurea. J Pediatr Hematol Oncol.

[23] Bjorner JB (2007). Developing tailored instruments: item banking and computerized adaptive assessment. Qual Life Res.

[24] Reeve BB (2007). Psychometric evaluation and calibration of health-related quality of life item banks: plans for the Patient-Reported Outcomes Measurement Information System (PROMIS). Med Care.

[25] Keller S, Yang M. Development of an "R"-Based Computer Adaptive Assessment of Health Based on Patient Reports of their Functioning and Wellbeing. Poster presented at: The 119th Annual American Psychological Association Convention; Aug 4-7 2011; Washington, D.C.

[26] Patrick DL, Deyo RA (1989). Generic and disease-specific measures in assessing health status and quality of life. Med Care.

[27] Kantz ME (1992). Methods for assessing condition-specific and generic functional status outcomes after total knee replacement. Med Care.

[28] Fried TR (2008). Views of older persons with multiple morbidities on competing outcomes and clinical decision-making. J Am Geriatr Soc.

[29] Gliklich RE, Hilinski JM (1995). Longitudinal sensitivity of generic and specific health measures in chronic sinusitis. Qual Life Res.

[30] Rothrock NE (2010). Relative to the general US population, chronic diseases are associated with poorer health-related quality of life as measured by the Patient-Reported Outcomes Measurement Information System (PROMIS). J Clin Epidemiol.

[31] Weaver KE (2010). Mental and physical health-related quality of life among US cancer survivors: population estimates from the 2010 National Health Interview Survey. Cancer epidemiology, biomarkers & prevention : a publication of the American Association for Cancer Research, cosponsored by the American Society of Preventive Oncology.

[32] Bessette L (1998). Comparative responsiveness of generic versus disease-specific and weighted versus unweighted health status measures in carpal tunnel syndrome. Med Care.

[33] Wong CK (2013). Condition-specific measure was more responsive than generic measure in colorectal cancer: all but social domains. J Clin Epidemiol.

[34] Walsh TL (2003). Is a condition-specific instrument for patients with low back pain/leg symptoms really necessary? The responsiveness of the Oswestry Disability Index, MODEMS, and the SF-36. Spine (Phila Pa 1976).

[35] Papuga MO (2014). Validation of GAITRite and PROMIS as high-throughput physical function outcome measures following ACL reconstruction. J Orthop Res.

[36] Panepinto JA (2013). PedsQL^TM^ sickle cell disease module: feasibility. reliability and validity. Pediatr Blood Cancer.

[37] Roland M, Morris R (1983). A study of the natural history of back pain. Part I: development of a reliable and sensitive measure of disability in low-back pain. Spine (Phila Pa 1976).

[38] Ren XS (2005). The role of generic and disease-specific measures of physical and role functioning in assessing patient outcomes: a longitudinal study. J Ambul Care Manage.

[39] US Food and Drug Administration. Guidance for Industry: Patient-Reported Outcome Measures: Use in Medical Product Development to Support labeling Claims Available from: https://www.fda.gov/downloads/drugs/guidances/ucm193282.pdf. Accessed 25 Apr 2017. 10.1186/1477-7525-4-79 PMC1629006 17034633

[40] Guyatt GH (1987). A measure of quality of life for clinical trials in chronic lung disease. Thorax.

[41] Sprangers MA (1996). The European Organization for Research and Treatment of Cancer breast cancer-specific quality-of-life questionnaire module: first results from a three-country field study. J Clin Oncol.

[42] de Boer AG (1996). Quality of life in patients with Parkinson's disease: development of a questionnaire. J Neurol Neurosurg Psychiatry.

[43] Marks GB, Dunn SM, Woolcock AJ (1992). A scale for the measurement of quality of life in adults with asthma. J Clin Epidemiol.

[44] Duncan PW (1999). The stroke impact scale version 2.0: evaluation of reliability, validity, and sensitivity to change. Stroke.

[45] Hung M (2012). New paradigm for patient-reported outcomes assessment in foot & ankle research: computerized adaptive testing. Foot Ankle Int.

[46] Olino TM (2013). Comparisons across depression assessment instruments in adolescence and young adulthood: an item response theory study using two linking methods. J Abnorm Child Psychol.

[47] Rose M (2008). Evaluation of a preliminary physical function item bank supported the expected advantages of the Patient-Reported Outcomes Measurement Information System (PROMIS). J Clin Epidemiol.

[48] Choi SW (2010). Efficiency of static and computer adaptive short forms compared to full-length measures of depressive symptoms. Qual Life Res.

[49] Bender MA, Seibel GD. Sickle cell disease. 2003, Sep 15 [updated 2014 Oct23] In: Pagan RA, et al. editors. GeneReviews® [Internet]. Seattle (WA): University of Washington, Seattle; 1993-2016.

[50] Steinberg MH (2008). Sickle cell anemia, the first molecular disease: overview of molecular etiology, pathophysiology, and therapeutic approaches. Scientific World J.

[51] Steinberg MH (2005). Predicting clinical severity in sickle cell anaemia. Br J Haematol.

[52] Sebastiani P (2007). A network model to predict the risk of death in sickle cell disease. Blood.

[53] Akinola NO (2009). The import of abdominal pain in adults with sickle cell disorder. West Afr J Med.

[54] Ballas SK (2001). Sickle cell disease: current clinical management. Semin Hematol.

[55] Sebastiani P (2010). Genetic modifiers of the severity of sickle cell anemia identified through a genome-wide association study. Am J Hematol.

[56] Steinberg MH, Adewoye AH (2006). Modifier genes and sickle cell anemia. Curr Opin Hematol.

[57] Mayer ML (2003). Hospital resource utilization among patients with sickle cell disease. J Health Care Poor Underserved.

[58] Loureiro MM (2009). Factors associated with hospital readmission in sickle cell disease. BMC Blood Disord.

[59] Dabari S, et al. Severe painful vaso-occlusive crises and mortality in contemporary adult sickle cell anemia cohort study. PLoS One. 2013;8(11):e79923. 10.1371/journal.pone.0079923 PMC3818240 24224021

[60] Audard V (2010). Acute kidney injury in sickle patients with painful crisis or acute chest syndrome and its relation to pulmonary hypertension. Nephrol Dial Transplant.

[61] Frei-Jones MJ (2009). Risk factors for hospital readmission within 30 days: a new quality measure for children with sickle cell disease. Pediatr Blood Cancer.

[62] Carroll CP (2009). The course and correlates of high hospital utilization in sickle cell disease: evidence from a large, urban Medicaid managed care organization. Am J Hematol.

[63] Smith WR (2008). Daily assessment of pain in adults with sickle cell disease. Ann Intern Med.

[64] Dampier C (2004). Vaso-occlusion in children with sickle cell disease: clinical characteristics and biologic correlates. J Pediatr Hematol Oncol.

[65] Vick LR (2009). Partial splenectomy prevents splenic sequestration crises in sickle cell disease. J Pediatr Surg.

[66] Halabi-Tawil M (2008). Sickle cell leg ulcers: a frequently disabling complication and a marker of severity. Br J Dermatol.

[67] Nolan VG (2006). Sickle cell leg ulcers: associations with haemolysis and SNPs in Klotho, TEK and genes of the TGF-beta/BMP pathway. Br J Haematol.

[68] Klings ES (2006). Abnormal pulmonary function in adults with sickle cell anemia. Am J Respir Crit Care Med.

[69] Gill FM (1995). Clinical events in the first decade in a cohort of infants with sickle cell disease. Cooperative Study of Sickle Cell Disease. Blood.

[70] Cheng L (2003). Health related quality of life in pregeriatric patients with chronic diseases at urban, public supported clinics. Health Qual Life Outcomes.

[71] Wensing M (2001). Functional status, health problems, age and comorbidity in primary care patients. Qual Life Res.

[72] Michelson H (2000). Multiple chronic health problems are negatively associated with health related quality of life (HRQoL) irrespective of age. Qual Life Res.

[73] Meyer HH (1951). Methods for scoring a check-list type rating scale. J Appl Psychol.

[74] Bland AC (2005). The psychometric properties of five scoring methods applied to the script concordance test. Acad Med.

[75] Ware JE, Sherbourne CD (1992). The MOS 36-item short-form health survey (SF-36). I. Conceptual framework and item selection. Med Care.

[76] Hays RD (1993). The RAND 36-Item Health Survey 1.0.. Health Econ.

[77] Jones N (2004). The Medicare Health Outcomes Survey program: overview, context, and near-term prospects. Health Qual Life Outcomes.

[78] Dampier C (2011). Health-related quality of life in adults with sickle cell disease (SCD): a report from the comprehensive sickle cell centers clinical trial consortium. Am J Hematol.

[79] Levenson JL (2008). Depression and anxiety in adults with sickle cell disease: the PiSCES project. Psychosom Med.

[80] Liu H (2010). Representativeness of the patient-reported outcomes measurement information system internet panel. J Clin Epidemiol.

[81] Cella D (2010). Initial item banks and first wave testing of the Patient-Reported Outcomes Measurement Information System (PROMIS) network: 2005–2008. J Clin Epidemiol.

[82] Cronbach L (1951). Coefficient alpha and the internal structure of tests. Psychometrika.

[83] Abdi H (2007). Bonferroni and Šidák corrections for multiple comparisons, in encyclopedia of measurement and statistics.

[84] Liang MH (1985). Comparative measurement efficiency and sensitivity of five health status instruments for arthritis research. Arthritis Rheum.

[85] Keller SD (1999). The SF-36 Arthritis-Specific Health Index (ASHI): II. Tests of validity in four clinical trials. Med Care.

[86] Cohen J (1988). Statistical power analysis for the behavioral sciences.

[87] Nunnally JC (1967). Psychometric theory.

[88] Streiner DL (2003). Starting at the beginning: an introduction to coefficient alpha and internal consistency. J Pers Assess.

[89] Kaplan RM, Saccuzzo DP (2013). Pscyhological testing: principles, applications and issues. Eighth ed.

[90] Nunnally JC, Bernstein IH (1994). Psychometric theory.

[91] Vichinsky E (2010). Neuropsychological dysfunction and neuroimaging adult sickle cell anemia study group. Neuropsychological dysfunction and neuroimaging abnormalities in neurologically intact adults with sickle cell anemia. JAMA.

[92] Ballas SK, et al. Beyond the definitions of the phenotypic complications of sickle cell disease: an update on management. The Scientific World J. 2012. Published online 2012 Aug 1. 10.1100/2012/949535 PMC3415156 22924029

[93] Tulsky DS (2015). Methodology for the development and calibration of the SCI-QOL item banks. J Spinal Cord Med.

[94] Keller S, Yang M (2015). Disease-specific calibrations or disease-specific content: which is responsible for the enhanced sensitivity of condition-specific measures compared to generic measures?. Qual Life Res.

[95] Centers for Disease Control and Prevention. Registry and Surveillance System for Hemoglobinopathies Pilot Project (RuSH). Available from: http://www.cdc.gov/ncbddd/sicklecell/freematerials.html.

[96] Feliu MH (2011). Neurocognitive testing and functioning in adults sickle cell disease. Hemoglobin.

[97] Cook KF (2012). A PROMIS fatigue short form for use by individuals who have multiple sclerosis. Qual Life Res.

[98] Kratz AL (2016). The PROMIS FatigueFM Profile: a self-report measure of fatigue for use in fibromyalgia. Qual Life Res.

[99] Choi SW (2009). Firestar: computerized adaptive testing simulation program for polytomous item response theory models. Appl Psychol Measur.

[100] Jaeschke R (1989). Measurement of health status: ascertaining the minimally clinically important difference. Cont Clin Trials.

